# The use of remdesivir outside of clinical trials during the COVID-19 pandemic

**DOI:** 10.1186/s40545-020-00258-8

**Published:** 2020-09-21

**Authors:** Vesa Halimi, Armond Daci, Nevenka Ridova, Irina Panovska-Stavridis, Milena Stevanovic, Venko Filipce, Aleksandar Dimovski, Aleksandra Grozdanova

**Affiliations:** 1 Faculty of Pharmacy, University Ss. Cyril and Methodius University, Skopje, North Macedonia; 2 Department of Pharmacy, Faculty of Medicine, University of Prishtina, Prishtina, Kosovo; 3 University Clinic of Hematology, Medical Faculty, University Ss. Cyril and Methodius University, Skopje, North Macedonia; 4 University Clinic of Infection Diseases and Febrile Conditions, Medical Faculty, University Ss. Cyril and Methodius, Skopje, North Macedonia; 5 University Clinic for Neurosurgery, Medical Faculty, University Ss. Cyril and Methodius, Skopje, North Macedonia

**Keywords:** COVID-19, Remdesivir, Clinical practice, Clinical trials, Expanded access, Compassionate use, Early access scheme, Emergency use, Conditional approval

## Abstract

With a scientific background from filoviruses, paramyxoviruses, SARS-CoV, and MERS-CoV, remdesivir entered into the COVID-19 battle to become one of the favorable therapeutic candidates with potential antiviral activity in the treatment of this disease. Globally, remdesivir was accessed and investigated through clinical research (clinical trials) and clinical practice (compassionate use, expanded access, early access scheme, and emergency use). Currently, remdesivir approval status differs between states. This paper aims to review and analyze regulatory approaches for accessing and investigating remdesivir, by communicating regulatory variability between countries in terms of terminology, modalities, and protocols.

## Introduction

The undisputable medical need to find an effective therapeutic in the middle of COVID-19 pandemic, aligned regulatory authorities, clinicians, researchers, and manufacturers to consider using unapproved therapeutics or unapproved indications of already approved therapeutics with potential antiviral activity [1]. The main goal of finding an effective therapy is not only to determine the clinical efficacy and safety but also to determine the treatment duration, sensitive patients, and to ensure equitable patient access. As clinical trials began to show their preliminary outcomes, the established regulatory approaches to ensure the access to potential COVID-19 therapeutics have been reviewed, and as a consequence, some therapeutics have been revoked and are not recommended for use anymore [2, 3].

For instance, the rapid approval of emergency use of hydroxychloroquine and chloroquine in the USA on 28 March 2020 [4], consequently revoked on 15 June 2020 [2], for safety and efficacy reasons, can be justified to some extent for the fact that these two antimalarial drugs had an already known safety profile in their approved indication(s). Also, the availability of scientific evidence in terms of safety and efficacy prompted FDA to decide that the potential benefits of hydroxychloroquine and chloroquine outweigh their potential risks, considering that no effective therapy was approved at that time [5]. On the other hand, several immunomodulating therapeutics like dexamethasone [6, 7], interleukin-1 inhibitors [8, 9], interleukin-6 inhibitors [10–12], interferon-beta [13], Bruton’s tyrosine kinase inhibitors [14], Janus kinase inhibitors [15], have raised hope in the treatment of COVID-19. But with regard to remdesivir, some of the pertinent questions include how was remdesivir accessed in patients outside of clinical trials? How regulatory authorities and Gilead have communicated these regulatory approaches to clinicians? How the preliminary results of RCTs have affected the expansion of remdesivir’s use in clinical practice, and later, its approval?

### Aims and methods to evaluate data on remdesivir

The main objective of this paper is to elaborate and evaluate regulatory approaches for the use of remdesivir outside of clinical trials. We analyzed and relied on the scientific opinions and recommendations for remdesivir published by European Medicines Agency (EU-EMA) [16], Food and Drug Administration (US-FDA) [4] and Medicines and Healthcare products Regulatory Agency (UK-MHRA) [17]. We compared and analyzed the data with the Gilead’s protocol for compassionate use that is available on the website of the Italian Regulatory Agency (AIFA) [18]. The regulatory guidance and materials [16, 19–30] (Tables 1 and 2) were compared with peer-reviewed original articles considering compassionate use of remdesivir [31].

**Table 1:** Legal basis, terminologies, and regulatory evidence for compassionate use, expanded access, and for early access scheme in COVID-19

Regulatory agency	Legal basis	Terminology and other modalities	Additional regulatory evidence for the use remdesivir in COVID-19, outside of clinical trials
Food and Drug Administration, FDA, USA	21 CFR 312 via 21CFR 312.300, 21CFR 312.305, 21CFR 312.310, 21CFR 312.315, 21CFR312.320	Expanded access for individual patients, in emergency use, for intermediate-size population, for widespread treatment use.	NCT04323761 treatment IND/protocol [19]; NCT04302766 treatment IND/protocol [20];
Medicines and Healthcare Products Regulatory Agency (MHRA), UK	Human Medicines Regulations 2012 (SI 2012/1916)	Early access to medicines intended for more patients	-Treatment protocol for healthcare professionals_ EAMS 11972/0002 remdesivir 100 mg concentrate for solution for infusion [21]. -Treatment protocol for healthcare professionals_EAMS 11972/0001 remdesivir 100 mg powder for concentrate for solution for infusion [22]; -Treatment protocol for patients_EAMS 11972/0001 remdesivir 100 mg powder for concentrate for solution for infusion [23]; -Treatment protocol for patients_EAMS 11972/0002 remdesivir 100 mg concentrate for solution for infusion [24]; -Treatment protocol on the pharmacovigilance system -Information for medical directors [25]; -Early Access to Medicines Scientific Opinion-Public Assessment Report [26];
European Medicines Agency EMA, European Union	Article 83 of Regulation (EC) No. 726/2004	Compassionate use is intended for a group of patients, although the approval, terminology and modalities are left within the remit of the National European Agencies.	-Conditions of use, conditions for distribution and patients targeted, and conditions for safety monitoring addressed to member states for remdesivir available for compassionate use [16]; -Summary on compassionate use [27];

**Table 2:** Legal basis and regulatory evidence for emergency use of remdesivir in the USA

Emergency use of remdesivir	Legal basis	Regulatory evidence issued by FDA
USA	Section 564 of the FD&C Act amended: -By the Project Bioshield Act of 2004; -By the Pandemic and All-Hazards Preparedness Reauthorization Act of 2013 (PAHPRA), the 21st Century Cures Act of 2016, and Public Law 115-92 of 2017	• Fact Sheet for Health Care Providers [28]; • Fact sheet for patients and parent/caregivers Spanish [29]; • Frequently asked questions on the emergency use authorization for remdesivir for certain hospitalized COVID-19 patients [30];

Two expanded access trials NCT04323761 [19] and NCT04302766 [20], as well as RCTs including NCT04257656 [32], NCT04292899 [33], NIAID trial [34], Inserm discovery trial, solidarity trial, and peer-reviewed articles automatically indexed to these trials, were analyzed [32–34]. As a support, we used the Gilead’s formal website to evaluate communication towards the public [35].

### The access to remdesivir outside of clinical trials

Based on the in vitro and animal studies from SARS-CoV and MERS-CoV as well as phase 1 clinical trials in healthy volunteers in EBOV, Gilead recommended the dosage and duration of treatment for remdesivir as 200 mg on day 1 followed by 100 mg from day 2 to 10 depending on the clinical assessment [36]. The access of remdesivir in COVID-19 pandemic was made possible through several regulatory approaches within the scope of clinical research and clinical practice [35, 37]. The primary suitable approach for accessing and investigating remdesivir were several clinical trials, on which patients within the eligibility criteria would be monitored properly and reliable and interpretable safety and efficacy data would be gathered. Meanwhile, patients who were not eligible to be enrolled in clinical trials would get access to remdesivir through compassionate use (expanded access, early access scheme) and emergency use [16, 26, 28] as given in Table 3. The approval of these regulatory approaches outside of clinical trials did not mean that remdesivir was safe and effective, and it was neither a formal approval nor a commitment for approval and not an authorization to provide remdesivir to patients.

**Table 3:** The use of remdesivir in clinical practice of the UK, Europe, and the USA

The use of remdesivir in COVID-19 outside of clinical trials: compassionate use, expanded access, early scheme, and emergency use				
➢ Primary objective: increase access for COVID-19 patients not eligible to be enrolled in clinical trials ➢ Secondary objective: safety Remdesivir 100 mg available in two pharmaceutical forms: 1.Concentrate for solution for infusion 2. Powder for concentrate for solution for infusion				
**UK**	**Europe**	**USA**		
**Informed consent**				**No informed consent**
**No control group**				
**Early access to medicines scheme**	**Compassionate use**	**Expanded access** **NCT04323761** **Treatment IND/Protocol**	**Expanded access** **NCT04302766 Treatment IND/Protocol**	**Emergency use**
**Inclusion criteria**				
**-Adults, adolescents, and children** ≥ 12 years -At least 40 kg -Suspected or diagnosed COVID-19 patients requiring or not invasive ventilation and/or ECMO. -Pregnancy—to be used with caution	**-Adults, adolescents, and children** ≥ 12 years -At least 40 kg -Suspected or diagnosed COVID-19 patients requiring or not requiring invasive ventilation and/or ECMO.	**Adults, adolescents, and children** ≥ 12 years -At least 40 kg suspected or diagnosed COVID-19 patients.	**Adults, adolescents, and children** -Department of defense’s personnel -Available for clinical follow-up for duration of the treatment and follow-up reports.	**-Adults, adolescents, and children from 0 year old** -At least 3.5 kg -suspected or diagnosed COVID-19 patients requiring or not invasive ventilation and/or ECMO. -Pregnancy—to be used with caution
**Exclusion criteria**				
-Children < 12 -ALT ≥ 5 ULN; -eGFR < 30; -Hypersensitivity	-Children < 12 -ALT ≥ 5 ULN; -eGFR < 30; -Hypersensivity -Multi-organ failure; -Use of more than 1 pressor shock -Hypersensivity -Pregnancy	-Children < 12 -ALT > 5 ULN -ALT > 3 and bilirubin > 2 ULN -eGFR < 30; -Multiorgan failure; -Use of more than 1 pressor shock -Hypersensivity -Pregnancy -Patients requiring venous-arterial ECMO (v-a)	ALT, AST ≥ 5 ULN; -eGFR < 30; -Hypersensivity; -Concomitant antiviral therapy (Kaltera) -Anticipated transfer to another hospital that is not a study site within 72 h. -Pregnancy	-ALT ≥ 5 ULN;-eGFR < 30; -Hypersensitivity
**Dosage and dosing regimen**				
-200 mg on day 1 -100 mg starting on day 2	-200 mg on day 1 -100 mg starting on day 2	**NA**	**NA**	Adults -200 mg on day 1 -100 mg starting on day 2 Pediatric patients 100 mg, only powder for concentrate for solution for infusion -5 mg/kg on day 1 -2.5 mg/kg starting on day 2
-Patients not requiring invasive ventilation will be given remdesivir every day for a total of 5 days with possible extension to 10 days. -Patients requiring invasive ventilation and/or on ECMO will be given remdesivir every day for a total of 10 days.	-Patients not requiring invasive ventilation will be given remdesivir every day for a total of 5 days with possible extension to 10 days. -Patients requiring invasive ventilation and/or on ECMO will be given remdesivir every day for a total of 10 days.	**NA**		-Patients not requiring invasive ventilation will be given remdesivir every day for a total of 5 days with possible extension to 10 days. -Patients requiring invasive ventilation and/or on ECMO will be given remdesivir every day for a total of 10 days.

When no data were published in the relevant database, the NA choice was used. Since in COVID-19 breastfeeding was not recommended and should be stopped due to the lack of adequate and controlled clinical evidence, we did not include this choice either in the inclusion or in exclusion criteria. ULN-upper limit normal. The expanded access NCT04323761/Treatment IND/Protocol has 268 locations located in the USA, Europe, Australia, Israel, and Canada and to date, it is still active

The first individual compassionate use request for remdesivir was received on January 2020, to treat a 35-year-old male COVID-19 patient in the USA [38], whereupon this approach was initiated and implemented by many other countries and in a larger number of patients [19]. The European Medicines Agency on 3 April 2020 provided not binding scientific recommendations and opinions for conditions of the use of remdesivir outside of clinical trials, requested from Greece, Estonia, Romania, and the Netherlands, recommending the target population, dosage, treatment duration, and a summary of the totality of the evidence [36]. Compassionate use of remdesivir in the EU was approved by national regulatory authorities of Switzerland, Spain, Slovenia, Hungary, Iceland, Greece, Italy, Netherlands, Portugal, Romania, Slovakia, Netherlands, Austria, Belgium, Germany, France, Estonia, and Cyprus [19]. This authorization was designated to treat a target group of COVID-19 patients not qualified for enrollment in clinical trials, for which European regulators would strictly review and track the safety data of remdesivir, rather than efficacy data [39]. Although most countries have similar regulatory requirements for enrollment to the compassionate use programs, there is no one-size-fits-all rule in terms of the legal basis, regulatory requirements, and terminology as given in Table 1. Compassionate use of Europe corresponds with expanded access in the USA designated and approved to be used for the treatment of individual patients, including in emergency use, for medium-size patent population and widespread use [40]. Hence, the compassionate use is not defined as a regulatory terminology by the Food and Drug Administration (FDA) [40]. Compassionate use of remdesivir in Europe is not designated for individual patients, because this approach falls within the remit of Article 5 of Directive 2001/83/EC also known as “Named Patient Basis,” applicable in emergencies [41]. Also, two expanded access trials such as the Gilead’s NCT04323761 [19] with 268 study locations to date, and the US army’s NCT04302766 [20] of remdesivir do not have enough public data. This is especially in the context of study design and interventions to be further interpreted.

However, from Gilead’s perspective, there are two terminologies and modalities such as “compassionate use” designated for individual patients and “expanded access” designated for a group of patients, stating that the manufacturer only accepts individual requests for pregnant women and children less than 18 years of age [35]. This is due to the process of transitioning in expanded access programs and the limited supply of the drug [35]. From the published original article of remdesivir’s compassionate use on 10 April 2020, we have seen that there have been several issues not only in the methodology, study design, but also in the implementation of this program in clinics across USA, Canada, Japan, and the EU [31]. From 25 January to 7 March, the data of 53 patients requiring invasive mechanical ventilation and ECMO were analyzed through this approach and clinical improvement was noted in 36 patients and a non-comprehensive safety report was available [31].

### The influence of results of clinical trials in expanding access to remdesivir

Compared to other potential drugs for treating COVID-19, remdesivir was accessed and investigated in fewer clinical trials. The multicenter double-blinded phase 3 RCT with a study design same as the National Institute of Allergy and Infectious Diseases (NIAID) trial (NCT04257656) reported inconclusive data due to the premature termination of COVID-19 outbreak in Hubei Province of China and the low-enrollment of patients [32]. Meanwhile, the open-label phase 3 RCT NCT04292899 published on 27 May 2020, did not show a significant difference between the 5-day course and 10-day course for COVID-19 patients not requiring invasive mechanical ventilation or ECMO in the baseline, while patients who would need invasive mechanical ventilation or ECMO, the treatment duration might be extended to 10 days [33]. Further interpretation of this trial would be of great significance, considering the limited medical supply and the high cost of remdesivir.

On the other hand, the National Institute of Allergy and Infectious Diseases (NIAID) double-blinded placebo trial NCT04280705, reported that remdesivir was superior to placebo in speeding up the recovery time in COVID-19 patients [34]. Remdesivir is enrolled also in the WHO solidarity clinical trial and INSERM discovery clinical trial. Nevertheless, there were still issues and gaps to be elucidated especially in terms of death rates, virus load data, and safety aspects [32–34]. However, from these preliminary data, the Food and Drug Administration (FDA) on 1 May 2020, approved its emergency use for patients at all ages requiring or not requiring invasive ventilation or ECMO [4]. Consequently, the European Medicines Agency (EMA) on 11 May 2020 revised the recommendations for compassionate use of remdesivir by extending the inclusion criteria with the addition of patients who do not require invasive ventilation to be treated for a period of 5 days through 10 days [16]. The Medicines and Healthcare products Regulatory Agency (MHRA) on 26 May 2020 approved early access scheme of remdesivir for patients of ≥ 12 years requiring or not requiring invasive mechanical ventilation [17]. Since most of the RCTs excluded pregnant women and breastfeeding, the only therapeutic option for this group of patients was the use of remdesivir outside of clinical trials, although the EMA did not agree to use remdesivir in pregnant women at the beginning [16].

### Conditional approval and further implications

In response to the current medical need for an effective therapeutic in context of public health crisis caused by COVID-19, the European Medicines Agency (EMA) on 30 April 2020 commenced a rolling review of the totality of evidence for remdesivir, as a potential therapeutic in the treatment of COVID-19 [42]. During this review, the EMA assessed the scientific dossier comprising of administrative, quality, preclinical, and clinical data proposed by Gilead and the available published literature and studies [43].

On 25 June 2020, the European Medicines Agency (EMA) issued a positive recommendation for the conditional approval of remdesivir in the European Union, although its safety and efficacy profile was not entirely elucidated [44]. Nevertheless, the EMA laid down explicit obligations in terms of quality, safety, and efficacy to be provided from Gilead, within a given deadline annexed at the European Public Assessment Report of remdesivir [43]. The approval of remdesivir by the European Union on 3 July 2020 was mainly influenced by the outcomes from the pivotal NIAID trial [43]. Under this approval, remdesivir will be available only for pneumonia COVID-19 patients (12 years and older, weighing at least 40 kg) requiring oxygen support in an initial dose of 200 mg on day 1 followed by 100 mg once daily on day 2–10 [44]. Soon after the issuance of conditional approval of remdesivir in the EU, the positive recommendation for early access scheme of remdesivir in the UK was withdrawn [45]. Mean while in the US, the emergency use authorization for remdesivir is still applicable [28]; Australia on 10 July 2020 issued a provisional approval for remdesivir after the recent approval from the EU, Japan, and Singapore [46]. To ensure equitable access for remdesivir in developing countries, the manufacturer Gilead is focused on advancing the manufacturing process and agreed to contract several generic developers to manufacture remdesivir [47]. To explore its potential to be used in the early stages of the disease and to avoid the hospitalization, the inhaled formulation of remdesivir is going to be evaluated in clinical trials [48]. Remdesivir is also under two clinical trials together with immunomodulators [49, 50] and under a clinical trial to evaluate the safety, tolerability, and pharmacokinetics in pediatric population [51]. Although on 10 July 2020, Gilead announced new additional information for remdesivir [52], still there is a need to accumulate more safety and efficacy data for the use of remdesivirespecially in children, pregnant women, breastfeeding, and patients with comorbidities.

## Conclusion

COVID-19 pandemic has influenced large clinical trials to be conducted. As for the use of remdesivir outside of clinical trials, the attitudes of regulatory authorities across countries have been different from one to another, either in regulatory approvals or in protocols. Preliminary results of RCTs (mainly the NIAID trial) have altered the opinion of regulatory authorities on remdesivir, either by recommending or approving its use outside of clinical trials. Only after the clinical endpoints and other crucial characteristics of RCTs will be analyzed and interpreted, and further trials will generate more data about specific population, we will be able to see if regulatory authorities have acted well or otherwise. It is imperative to educate clinicians regarding regulatory mechanisms for early access of investigational drugs so that they understand the possibilities that their patients have in unmet medical situations.

## Data Availability

Not applicable

## Abbreviations

EMA: European Medicines Agency
FDA: Food and Drug Administration
MHRA: Medicines and Healthcare Products Regulatory Agency
EBOV: Ebola virus
RCT: Randomized clinical trials
SARS-CoV: Severe acute respiratory syndrome coronavirus
MERS-CoV: Middle East respiratory syndrome coronavirus
ECMO: Extracorporeal membrane oxygenation
ECMO (v-v): Veno-venous extracorporeal membrane oxygenation
ECMO (v-a): Veno-arterial extracorporeal membrane oxygenation
ALT: Alanine aminotransferase
AST: Aspartate aminotransferase
eGFR: Estimated glomerular filtration rate
PAHPRA: the Pandemic and All-Hazards Preparedness Reauthorization Act
EAMS: Early access to medicines scheme
ULN: Upper limit normal
NIAID: National Institute of Allergy and Infectious Diseases

## References

[1] Halimi V, Daci A, Stojanovska S, Panovska-Stavridis I, Stevanovic M, Filipce V, et al. Current regulatory approaches for accessing potential COVID-19 therapies. J Pharm Policy Pract. 2020. 10.1186/s40545-020-00222-6 PMC7229878 32454981

[2] Food and Drug Administration (FDA). Letter revoking EUA for chloroquine phosphate and hydroxychloroquine sulfate. [Internet]. [cited 2020 July 19]. Available from: https://www.fda.gov/media/138945/download.

[3] Recovery statement. Statement from the Chief Investigators of the Randomised Evaluation of COVid-19 thERapY (RECOVERY) Trial on lopinavir-ritonavir [Internet]. [cited 2020 July 19]. Available from: www.recoverytrial.net.

[4] Food and Drug Administration (FDA). FDA COMBATING COVID-19 WITH THERAPEUTICS. [Internet]. [cited 2020 July 19]. Available from: https://www.fda.gov/media/136832/download.

[5] Food and Drug Administration (FDA). Request for emergency use authorization for use of chloroquine phosphate or hydroxychloroquine sulfate supplied from the strategic national stockpile for treatment of 2019 coronavirus disease [Internet]. [cited 2020 July 19]. Available from: https://www.fda.gov/media/136534/download.

[6] Villar J, Ferrando C, Martínez D, Ambrós A, Muñoz T, Soler JA, et al. Dexamethasone treatment for the acute respiratory distress syndrome: a multicentre, randomised controlled trial. Lancet Respir Med. 2020. 10.1016/S2213-2600(19)30417-5 32043986

[7] Horby P, Lim WS, Emberson J, Mafham M, Bell J, Linsell L, et al. Effect of Dexamethasone in Hospitalized Patients with COVID-19: Preliminary Report. medRxiv. 2020.

[8] Huet T, Beaussier H, Voisin O, Jouveshomme S, Dauriat G, Lazareth I, et al. Anakinra for severe forms of COVID-19: a cohort study. Lancet Rheumatol. 2020. 10.1016/S2665-9913(20)30164-8 PMC7259909 32835245

[9] Cavalli G, De Luca G, Campochiaro C, Della-Torre E, Ripa M, Canetti D, et al. Interleukin-1 blockade with high-dose anakinra in patients with COVID-19, acute respiratory distress syndrome, and hyperinflammation: a retrospective cohort study. Lancet Rheumatol. 2020. 10.1016/S2665-9913(20)30127-2 PMC7252085 32501454

[10] Liu T, Zhang J, Yang Y, Zhang L, Ma H, Li Z, et al. The Potential Role of IL-6 in Monitoring Coronavirus Disease 2019. SSRN Electron J. 2019:2020.

[11] Xu X, Han M, Li T, Sun W, Wang D, Fu B, et al. Effective treatment of severe COVID-19 patients with tocilizumab. Proc Natl Acad Sci U S A. 2020. 10.1073/pnas.2005615117 PMC7245089 32350134

[12] Toniati P, Piva S, Cattalini M, Garrafa E, Regola F, Castelli F (2020). Tocilizumab for the treatment of severe COVID-19 pneumonia with hyperinflammatory syndrome and acute respiratory failure: A single center study of 100 patients in Brescia.

[13] Hung IFN, Lung KC, Tso EYK, Liu R, Chung TWH, Chu MY, et al. Triple combination of interferon beta-1b, lopinavir–ritonavir, and ribavirin in the treatment of patients admitted to hospital with COVID-19: an open-label, randomised, phase 2 trial. Lancet. 2020. 10.1016/S0140-6736(20)31042-4 PMC7211500 32401715

[14] Treon SP, Castillo JJ, Skarbnik AP, Soumerai JD, Ghobrial IM, Guerrera ML, et al. The BTK inhibitor ibrutinib may protect against pulmonary injury in COVID-19-infected patients. Blood. 2020. 10.1182/blood.2020006288 PMC7243149 32302379

[15] Cao Y, Wei J, Zou L, Jiang T, Wang G, Chen L, et al. Ruxolitinib in treatment of severe coronavirus disease 2019 (COVID-19): A multicenter, single-blind, randomized controlled trial. J Allergy Clin Immunol. 2020. 10.1016/j.jaci.2020.05.019 PMC7250105 32470486

[16] European Medicines Agency (EMA). Annex I Conditions of use, conditions for distribution and patients targeted adressed to Member States Remdesivir Gilead. [Internet]. [cited 2020 Jul 19] Available online:https://www.ema.europa.eu/en/documents/other/conditions-use-conditions-distribution-patients-targeted-conditions-safety-monitoring-adressed_en-2.pdf.

[17] Medicines and Healthcare products Regulatory Agency (MHRA). MHRA issues a scientific opinion for the first medicine to treat COVID-19 in the UK - GOV.UK [Internet]. [cited 2020 Jul 1]. Available from: https://www.gov.uk/government/news/mhra-supports-the-use-of-remdesivir-as-the-first-medicine-to-treat-covid-19-in-the-uk.

[18] Agenzia Italiana del Farmaco (AIFA). Programmi di uso compassionevole – COVID-19 | Agenzia Italiana del Farmaco [Internet]. [cited 2020 Jul 12]. Available from: https://www.aifa.gov.it/programmi-di-uso-compassionevole-covid-19.

[19] ClinicalTrials.gov National Library of Medicine (US).Identifier NCT04323761. Expanded Access Treatment Protocol: Remdesivir (RDV; GS-5734) for the Treatment of SARS-CoV2 (CoV) Infection (COVID-19) - Full Text View - ClinicalTrials.gov [Internet]. [cited 2020 Jul 19]. Available from: https://www.clinicaltrials.gov/ct2/show/NCT04323761?term=remdesivir&type=Expn&cond=Covid-19&draw=2&rank=1.

[20] ClinicalTrials.gov National Library of Medicine (US).Identifier NCT04302766. Expanded Access Remdesivir (RDV; GS-5734^TM^) - Full Text View - ClinicalTrials.gov [Internet]. [cited 2020 Jul 19]. Available from: https://www.clinicaltrials.gov/ct2/show/NCT04302766.

[21] Medicines and Healthcare products Regulatory Agency(MHRA). EAMS Information for HCP [Remdesivir 100 mg concentrate for solution for infusion] EAMS 11972/0002 Remdesivir 100 mg concentrate for solution for infusion Early Access to Medicines Scheme-Treatment protocol-Information for healthcare professionals. [Internet]. [cited 2020 Jul 1] Available from: https://assets.publishing.service.gov.uk/government/uploads/system/uploads/attachment_data/file/887275/EAMS__11972_0002__TP__HCP_Solution.pdf.

[22] Medicines and Healthcare products Regulatory Agency (MHRA). EAMS Information for HCP [Remdesivir 100 mg powder for concentrate for solution for infusion]. [Internet]. [cited 2020 Jul 1] Available from: https://assets.publishing.service.gov.uk/government/uploads/system/uploads/attachment_data/file/887276/EAMS_11972_0001__TP_HCP_Powder.pdf.

[23] Medicines and Healthcare products Regulatory Agency (MHRA). EAMS Information for Patients [Remdesivir 100 mg powder for concentrate for solution for infusion] . [Internet]. [cited 2020 Jul 1] Available from: https://www.gov.uk/government/publications/early-access-to-medicines-scheme-eams-scientific-opinion-remdesivir-in-the-treatment-of-patients-hospitalised-with-suspected-or-laboratory-confirme/treatment-protocol-for-patients_eams-119720001-remdesivir-100-mg-powder-for-concentrate-for-solution-for-infusion.

[24] Medicines and Healthcare products Regulatory Agency (MHRA). EAMS information for patients [remdesivir 100 mg concentrate for solution for infusion] [Internet]. [cited 2020 Jul 1] Available from: https://assets.publishing.service.gov.uk/government/uploads/system/uploads/attachment_data/file/887278/EAMS_TP_for_Patients_11972_0002_Solution.pdf.

[25] Medicines and Healthcare products Regulatory Agency (MHRA). EAMS pharmacovigilance system [remdesivir 100 mg powder for concentrate for solution for infusion and remdesivir 100 mg concentrate for solution for infusion] EAMS 11972/0001 Remdesivir 100 mg powder for concentrate for solution for infusion EAMS 11972/0002 Remdesivir 100 mg concentrate for solution for infusion early access to medicines scheme-treatment protocol-information on the pharmacovigilance system and requirements for reporting safety data [Internet]. [cited 2020 Jul 1] Available from: https://assets.publishing.service.gov.uk/government/uploads/system/uploads/attachment_data/file/887279/EAMS_TP_on_PVS_11972_0001_and_11972_0002.pdf;.

[26] Medicines and Healthcare products Regulatory Agency (MHRA). Early access to medicines scientific opinion-public assessment report product remdesivir 100 mg powder for concentrate for solution for infusion remdesivir 100 mg concentrate for solution for infusion [Internet]. [cited 2020 Jul 1]. Available from: https://assets.publishing.service.gov.uk/government/uploads/system/uploads/attachment_data/file/897613/PAR_EAMS_11972_0001_AND_11972_0002__PAR.pdf.

[27] European Medicines Agency (EMA). Summary on compassionate use for Remdesivir Gilead [Internet]. [cited 2020 Apr 12]. Available from: https://www.ema.europa.eu/en/documents/other/summary-compassionate-use-remdesivir-gilead_en.pdf.

[28] Food and Drug Administration (FDA). Fact sheet for health care providers emergency use authorisation (EUA) of remdesivir (GS-5734^TM^) ). [Internet]. [cited 2020 Jul 4]. Available from: https://www.fda.gov/media/137566/download.

[29] Food and Drug Administration (FDA). Fact sheet for patients and parent/caregivers emergency use authorization (EUA) of remdesivir for coronavirus disease 2019 [Internet]. [cited 2020 Jul 4]. Available from: https://www.fda.gov/media/137565/download.

[30] Food and Drug Administration (FDA). Frequently asked questions on the emergency use authorization for remdesivir for certain hospitalized COVID-19 patients. [Internet]. [cited 2020 Jul 4]. Available from:https://www.fda.gov/media/137574/download.

[31] Grein J, Ohmagari N, Shin D, Diaz G, Asperges E, Castagna A, et al. Compassionate Use of Remdesivir for Patients with Severe Covid-19. N Engl J Med. 2020. 10.1056/NEJMoa2007016 PMC7169476 32275812

[32] Wang Y, Zhang D, Du G, Du R, Zhao J, Jin Y, et al. Remdesivir in adults with severe COVID-19: a randomised, double-blind, placebo-controlled, multicentre trial. Lancet. 2020. 10.1016/S0140-6736(20)31022-9 PMC7190303 32423584

[33] Goldman JD, Lye DCB, Hui DS, Marks KM, Bruno R, Montejano R, et al. Remdesivir for 5 or 10 Days in Patients with Severe Covid-19. N Engl J Med [Internet]. Massachusetts Medical Society; 2020 [cited 2020 Jun 5];NEJMoa2015301. Available from: http://www.nejm.org/doi/10.1056/NEJMoa2015301. 10.1056/NEJMoa2015301 PMC7377062 32459919

[34] Beigel JH, Tomashek KM, Dodd LE, Mehta AK, Zingman BS, Kalil AC, et al. Remdesivir for the treatment of Covid-19 — preliminary report. N Engl J Med [Internet]. Massachusetts Medical Society; 2020 [cited 2020 Jun 5];NEJMoa2007764. Available from: http://www.nejm.org/doi/10.1056/NEJMoa2007764.

[35] Gilead Sciences. Gilead Sciences Statement on Access to Remdesivir Outside of Clinical Trials [Internet]. [cited 2020 Jun 2]. Available from: https://www.gilead.com/news-and-press/company-statements/gilead-sciences-statement-on-access-to-remdesivir-outside-of-clinical-trials.

[36] European Medicines Agency (EMA). EMA provides recommendations on compassionate use of remdesivir for COVID-19 | European Medicines Agency [Internet]. [cited 2020 Jun 4]. Available from: https://www.ema.europa.eu/en/news/ema-provides-recommendations-compassionate-use-remdesivir-covid-19.

[37] European Medicines Agency. Treatments and vaccines for COVID-19 | European Medicines Agency [Internet]. [cited 2020 Apr 21]. Available from: https://www.ema.europa.eu/en/human-regulatory/overview/public-health-threats/coronavirus-disease-covid-19/treatments-vaccines-covid-19.

[38] Holshue ML, DeBolt C, Lindquist S, Lofy KH, Wiesman J, Bruce H, et al. First case of 2019 novel coronavirus in the United States. N Engl J Med. 2020. 10.1056/NEJMoa2001191 PMC7092802 32004427

[39] European Union; European Medicines Agency. Guideline on compassionate use of medicinal products, pursuant to Article 83 of Regulation (EC) No 726/2004. 2007; Available from: https://www.ema.europa.eu/en/documents/regulatory-procedural-guideline/guideline-compassionate-use-medicinal-products-pursuant-article-83-regulation-ec-no-726/2004_en.pdf;.

[40] Food and Drug Administration. Expanded access to investigational drugs for treatment use — questions and answers [Internet]. 2017. p. 1473–4. Available from: https://www.fda.gov/media/85675/download;.

[41] European Parliament and the Council of the European Union. Directive 2001/83/EC of the European Parliament and of the Council of 6 November 2001 on the Community code relating to medicinal products for human use [Internet]. Off. J. L 311 , 28/11/2001 P. 0067 - 0128; OPOCE; [cited 2020 Jun 9]. Available from: https://eur-lex.europa.eu/legal-content/EN/TXT/HTML/?uri=CELEX:32001L0083&from=EN.

[42] European Medicines Agency (EMA). EMA starts rolling review of remdesivir for COVID-19 | European Medicines Agency [Internet]. [cited 2020 Jun 12]. Available from: https://www.ema.europa.eu/en/news/ema-starts-rolling-review-remdesivir-covid-19.

[43] European Medicines Agency (EMA). European Public Assessment Report (EPAR) of Venklury (remdesivir) [Internet]. [cited 2020 Jun 9].Available from: www.ema.europa.eu/contact;.

[44] European Medicines Agency (EMA). First COVID-19 treatment recommended for EU authorisation | European Medicines Agency [Internet]. [cited 2020 Jul 19]. Available from: https://www.ema.europa.eu/en/news/first-covid-19-treatment-recommended-eu-authorisation.

[45] Medicines and Healthcare products Regulatory Agency (MHRA). [Withdrawn] Early access to medicines scheme (EAMS) scientific opinion: remdesivir in the treatment of patients hospitalised with suspected or laboratory-confirmed SARS-CoV-2 infection who meet the clinical criteria - GOV.UK [Internet]. [cited 2020 Jul 19]. Available from: https://www.gov.uk/government/publications/early-access-to-medicines-scheme-eams-scientific-opinion-remdesivir-in-the-treatment-of-patients-hospitalised-with-suspected-or-laboratory-confirme;.

[46] The Therapeutic Goods Administration (TGA). Australia’s first COVID treatment approved | Therapeutic Goods Administration (TGA) [Internet]. [cited 2020 Jul 19]. Available from: https://www.tga.gov.au/media-release/australias-first-covid-treatment-approved.

[47] Gilead Sciences. An Open Letter from Daniel O’Day, Chairman & CEO, Gilead Sciences [Internet]. [cited 2020 Jul 19]. Available from: https://stories.gilead.com/articles/an-open-letter-from-daniel-oday-june-29.

[48] Gilead Sciences. An Open Letter from Daniel O’Day, Chairman & CEO, Gilead Sciences [Internet]. [cited 2020 Jul 19]. Available from: https://stories.gilead.com/articles/an-open-letter-from-daniel-oday-june-22.

[49] U.S. National Library of Medicine Clinicaltrials.gov. A study to evaluate the efficacy and safety of remdesivir plus tocilizumab compared with remdesivir plus placebo in hospitalized participants with severe covid-19 pneumonia - full text view - ClinicalTrials.gov [Internet]. [cited 2020 Jul 19]. Available from: https://www.clinicaltrials.gov/ct2/show/NCT04409262?term=remdesivir&draw=3&rank=11.

[50] U.S. National Library of Medicine ClinicalTrials.gov. Adaptive COVID-19 Treatment Trial 2 (ACTT-2) - Full Text View - ClinicalTrials.gov [Internet]. [cited 2020 Jul 19]. Available from: https://www.clinicaltrials.gov/ct2/show/NCT04401579?term=remdesivir&draw=2&rank=13.

[51] U.S. National Library of Medicine ClinicalTrials.gov. Study to evaluate the safety, tolerability, pharmacokinetics, and efficacy of remdesivir (GS-5734^TM^) in participants from birth to < 18 years of age with coronavirus disease 2019 (COVID-19) - full text view - ClinicalTrials.gov [Internet]. [cited 2020 Jul 23]. Available from: https://clinicaltrials.gov/ct2/show/NCT04431453.

[52] Gilead Sciences. Gilead presents additional data on investigational antiviral remdesivir for the treatment of COVID-19 [Internet]. [cited 2020 Jul 19]. Available from: https://www.gilead.com/news-and-press/press-room/press-releases/2020/7/gilead-presents-additional-data-on-investigational-antiviral-remdesivir-for-the-treatment-of-covid-19.

